# Peak systolic velocity ratio for evaluation of internal carotid artery stenosis correlated with plaque morphology: substudy results of the ANTIQUE study

**DOI:** 10.3389/fneur.2023.1206483

**Published:** 2023-11-06

**Authors:** David Pakizer, Anna Vybíralová, Tomáš Jonszta, Martin Roubec, Michal Král, Vendelín Chovanec, Roman Herzig, Tomáš Heryán, David Školoudík

**Affiliations:** ^1^Faculty of Medicine, University of Ostrava, Ostrava, Czechia; ^2^Faculty of Health Sciences, Palacký University Olomouc, Olomouc, Czechia; ^3^Department of Radiology, University Hospital Ostrava, Ostrava, Czechia; ^4^Department of Neurology, Clinic of Neurology, University Hospital Ostrava, Ostrava, Czechia; ^5^Department of Neurology, University Hospital Olomouc, Olomouc, Czechia; ^6^Department of Radiology, University Hospital Hradec Kralove, Hradec Králové, Czechia; ^7^Department of Neurology, University Hospital Hradec Kralove, Hradec Kralove, Czechia; ^8^Department of Neurology, Faculty of Medicine in Hradec Králové, Charles University, Prague, Czechia; ^9^Department of Finance and Accounting, Silesian University in Opava, Opava, Czechia

**Keywords:** internal carotid artery, carotid stenosis – diagnosis, atherosclerotic plaque, duplex sonography, peak systolic velocity, computed tomography angiography

## Abstract

**Background:**

Accurate assessment of carotid stenosis severity is important for proper patient management. The present study aimed to compare the evaluation of carotid stenosis severity using four duplex sonography (DUS) measurements, including peak systolic velocity (PSV), PSV ratio in stenosis and distal to stenosis (PSV_ICA/ICA_ ratio), end-diastolic velocity (EDV), and B-mode, with computed tomography angiography (CTA), and to evaluate the impact of plaque morphology on correlation between DUS and CTA.

**Methods:**

Consecutive patients with carotid stenosis of ≥40% examined using DUS and CTA were included. Plaque morphology was also determined using magnetic resonance imaging. Spearman’s correlation and Kendall’s rank correlation were used to evaluate the results.

**Results:**

A total of 143 cases of internal carotid artery stenosis of ≥40% based on DUS were analyzed. The PSV_ICA/ICA_ ratio showed the highest correlation [Spearman’s correlation *r* = 0.576) with CTA, followed by PSV (*r* = 0.526), B-mode measurement (*r* = 0.482), and EDV (*r* = 0.441; *p* < 0.001 in all cases]. The worst correlation was found for PSV when the plaque was calcified (*r* = 0.238), whereas EDV showed a higher correlation (*r* = 0.523). Correlations of B-mode measurement were superior for plaques with smooth surface (*r* = 0.677), while the PSV_ICA/ICA_ ratio showed the highest correlation in stenoses with irregular (*r* = 0.373) or ulcerated (*r* = 0.382) surfaces, as well as lipid (*r* = 0.406), fibrous (*r* = 0.461), and mixed (*r* = 0.403; *p* < 0.01 in all cases) plaques. Nevertheless, differences between the mentioned correlations were not statistically significant (*p* > 0.05 in all cases).

**Conclusion:**

PSV, PSV_ICA/ICA_ ratio, EDV, and B-mode measurements showed comparable correlations with CTA in evaluation of carotid artery stenosis based on their correlation with CTA results. Heavy calcifications and plaque surface irregularity or ulceration negatively influenced the measurement accuracy.

## Introduction

The bifurcation of carotid arteries is one of the most common locations of atherosclerotic plaque build-up. Over time, it causes arterial stenosis with a prevalence of approximately 7–9% in the population (1, 2). Furthermore, large-vessel atherosclerotic cerebrovascular disease is an important cause of stroke. It is responsible for up to 15–20% of all ischemic strokes, while extracranial carotid atherosclerosis is the reason for approximately 7–18% of all first strokes (3, 4). Increasing degree of asymptomatic carotid artery stenosis is strongly related to the higher risk of ipsilateral stroke or transient ischemic attack. Therefore, slowing down the process of increasing volume of atherosclerotic plaque leading to increasing the severity of stenosis via lifestyle measures and/or medical treatment is desirable to prevent carotid occlusion or embolic pathogenesis of ipsilateral stroke (5). Carotid artery stenting (CAS) and carotid endarterectomy (CEA) are important successful invasive treatment methods mainly in patients with symptomatic severe extracranial internal carotid artery (ICA) stenosis with comparable outcomes that have been utilized over the last few decades (6–8).

Accurate assessment of carotid stenosis severity is important for proper patient management as a CEA/CAS treatment inclusion criterion (9, 10). Carotid stenosis can be measured using noninvasive imaging methods, such as duplex sonography (DUS), computed tomography (CT), and magnetic resonance imaging (MRI), or digital subtraction angiography (DSA) as an invasive method (11). Nevertheless, uncertainty associated with the use of different imaging and measurement methods can lead to inappropriate inclusion or exclusion of patients for invasive treatment (12). Therefore, a sufficiently accurate measurement of carotid stenosis is needed.

Carotid DUS is recommended for the initial and annual evaluation of patients with carotid atherosclerotic disease. It is currently the first-line diagnostic tool for detection and classification of internal carotid artery (ICA) stenosis severity that can be used for clinical decision-making (13). Measurement of peak systolic velocity (PSV), end-diastolic velocity (EDV), and morphological measurement of the arterial lumen in B-mode are the main duplex sonographic criteria for the evaluation of moderate-to-severe carotid stenoses. Nevertheless, ultrasound (US) techniques have their own limitations that influence the measurement in B-mode (e.g., location of bifurcation above the mandibular edge, tortuosity, severely calcified and echolucent lesions, and completely occluded carotid artery) or PSV measurement (e.g., Doppler angle, stenosis morphology, collaterals, nearly occluded artery, and atrial fibrillation) (14, 15). PSV ratio in stenosis and distal to stenosis (PSV_ICA/ICA_ ratio) could overcome some of these limitations.

The present study aimed to (1) measure correlations between the percentage of carotid stenosis evaluated by CT angiography (CTA) and DUS measurements based on PSV, PSV_ICA/ICA_ ratio, EDV, and B-mode measurement criteria; and (2) evaluate the influence of plaque morphology on these correlations.

## Methods

The present study was conducted in accordance with the ethical standards of the Ethics Committee of the University Hospital of Ostrava (approval no. 497/2017), Ethics Committee of the Faculty of Medicine, University of Ostrava (approval no. R1/2022), and the 1975 Declaration of Helsinki (as revised in 1983 and 2008). All patients provided written informed consent. The study was registered at ClinicalTrials.gov (ClinicalTrials.gov Identifier: NCT05390983).

The sample size for the multicentric quantitative observational study covered consecutive patients from the Atherosclerotic Plaque Characteristic Associated with a Progression Rate of the Plaque and a Risk of Stroke in Patients with the Carotid Bifurcation Plaque Study (ANTIQUE study; ClinicalTrials.gov Identifier: NCT02360137) examined by DUS and CTA. Consecutive patients were included based on the following criteria:

- Age 30–90 years;
- Carotid artery stenosis of ≥40% according to DUS examination in B-Mode (16);
- Self-sufficiency of patient, i.e., modified Rankin scale score of 0–2;
- Sufficient image quality of the carotid plaque on CTA and DUS;
- Signed informed consent.

Patients with contralateral carotid artery stenosis or occlusion, surgery, or stent implementation in cervical or intracranial arteries were excluded from analysis.

### Duplex sonography

**Figure 1:**
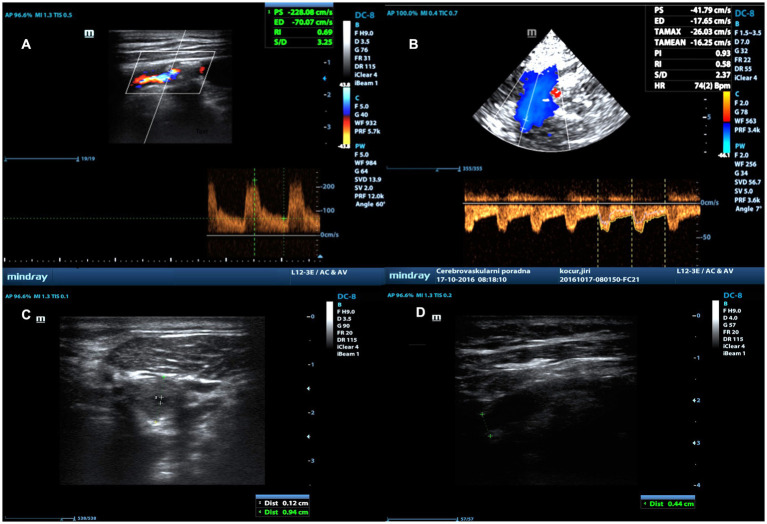
Measurement of percentage of stenosis using PSV_ICA/ICA_ ratio using duplex ultrasonography. Measurement of PSV in stenosis using linear probe **(A)**, PSV distal to stenosis using transcranial probe **(B)**, residual lumen in stenosis **(C)** and lumen diameter distal to stenosis **(D)** in B-Mode using linear probe.

DUS was performed on all patients at standard conditions during the baseline visit using the Mindray DC-8 expert US machine (Mindray, Shenzhen, China). The common carotid artery (CCA), proximal part of ICA, and external carotid artery were examined in both longitudinal and transverse planes in B-mode, color mode, and Doppler mode using a linear duplex probe L12-3E (3–12 MHz). The settings for the linear probe were standardized to optimize acquisition as follows: acoustic power (maximum); mechanical index (1.3); frame rate (20 fps); main frequency (9.0 MHz), harmonic frequencies (on); dynamic range (115 dB); iClear (4); iBeam (1); and depth and gain that were individually adjusted. The distal extracranial part of the ICA was examined using a transcranial duplex probe P4-2NE (1–4 MHz) with the following standardized settings: main frequency (1.5–3.5 MHz); depth (15.0 cm); gain (32 dB); frame rate (11 fps); dynamic range (55 dB); and iClear (4). The following parameters used to measure stenosis severity were recorded in all patients: maximal angle-corrected PSV in carotid stenosis; angle-corrected PSV 4 ± 0.5 cm distal to stenosis (measured using transcranial duplex probe) with calculated PSV_ICA/ICA_ ratio (in ICA stenosis and distal to stenosis); PSV in CCA with calculated PSV_ICA_/PSV_CCA_ ratio; EDV in and 4 ± 0.5 cm distal to stenosis (measured using transcranial duplex probe); EDV in CCA with calculated PSV_ICA_/EDV_CCA_ ratio; and B-mode measurements of residual lumen, arterial diameter in the region of stenosis, and arterial diameter distal to stenosis with calculation of degree of stenosis using the NASCET formula [Figure 1; (6)]. All parameters except PSV 4 ± 0.5 cm distal to stenosis were measured using a linear probe. The distance between the PSV distal to the stenosis measurement site and the stenosis site was measured using a caliper. All DUS examinations were performed by experienced certified neurosonologists (DŠ, RH, MK, and MR) prior to CTA and MRI examinations.

### Computed tomography angiography

**Figure 2:**
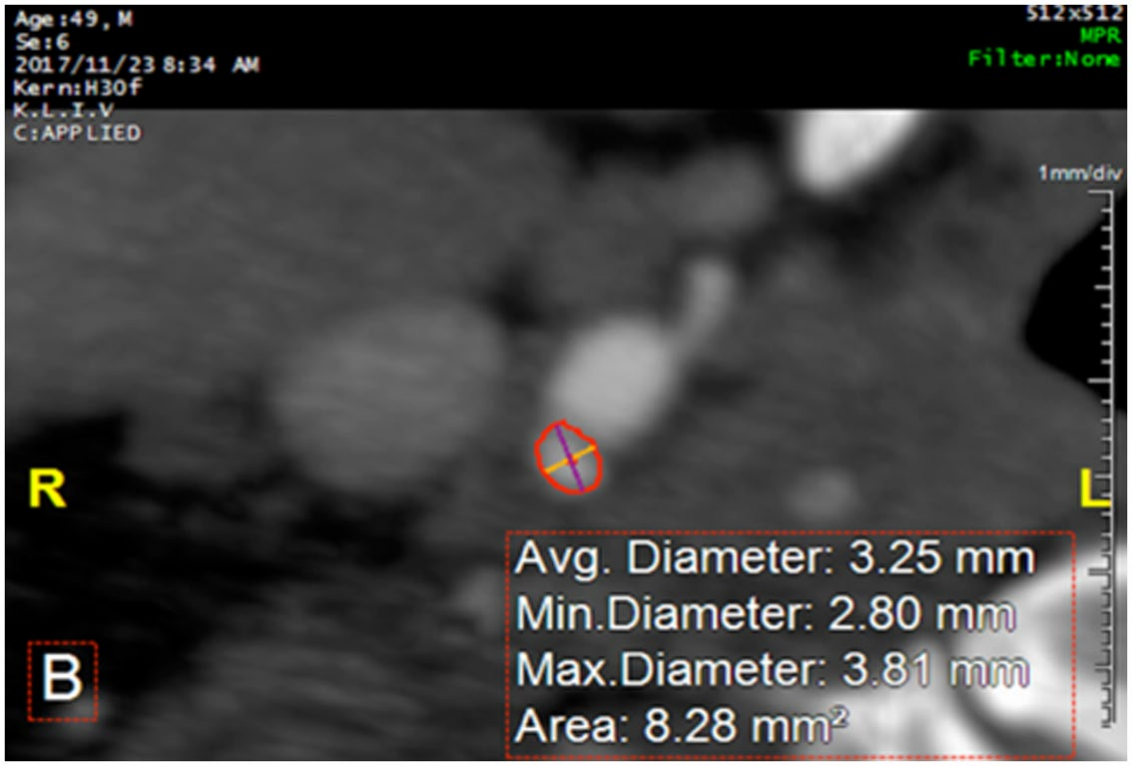
Measurement of the arterial lumen and percentage of stenosis using dedicated software on CTA.

All patients underwent multidetector CTA examinations of carotid arteries with Siemens Somatom Definition AS+ (Siemens Healthineers, Erlangen, Germany) and General Electric Discovery 750HD, Light Speed RT16 and Light Speed VCT (General Electric HeathCare Technologies, Chicago, IL, United States) devices. A total of 50–100 mL (depending on scan duration and patient weight) of Iomeron 400 (Bracco Imaging, Milan, Italy) or Ultravist 370 (Bayer HealthCare Pharmaceuticals LLC, Berlin, Germany) iodine contrast agents (CAs) were administered intravenously at a rate of 3–4 mL/s with an automatic injector through a 20 G cannula into a peripheral vein. Bolus triggering in the ascending aorta was used for planning the arterial phase during the examination. Multiplanar (axial plane, submillimeter sections) and maximum intensity projection (sagittal and coronal planes, 3–8-mm sections) reconstructions with a uniform window width (W) and center (L; W700/L200 HU) were assessed. The width and center of the window needed to be increased in several cases to reach optimal visualization (W700–1000/L200–400 HU). The degree of carotid artery stenosis was evaluated according to the NASCET formula (6) using dedicated software (TeraRecon Aquarius Viewer; TeraRecon, Durham, NC, USA; Figure 2).

### Magnetic resonance imaging

To better evaluate carotid plaque composition, an additional MRI examination of the neck was carried out on Siemens Magnetom Symphony 1.5T, Avanto 1.5T, Essenza 1.5T, and Skyra 3.0T (Siemens Healthineers, Erlangen, Germany) and Philips Ingenia 3.0T (Koninklijke Philips Electronics NV, Amsterdam, Netherlands) machines with a field of view centered on stenosis in the carotid bifurcation. Head/neck angiographic or cervical multichannel coils served as the receiving coils. The following four basic sequences were used in the examination protocol:

1. T1**-**weighted_TSE [turbo spin echo)_FS (fat suppressed) sequence, axial planes (time to echo (TE) 19 ms, time to repeat (TR) 600 ms; slice thickness (ST) 3 mm; matrix size 230 × 256; distance factor (gap) 0.3 mm; field of view (FOV) 256 mm; FOV phase 100%; turbo factor (TF) 2; number of excitations (NEX0 2; and sequence length 3:50 min).
2. The 3D_T1_MPRAGE (magnetization-prepared rapid gradient-echo) sequence, axial planes, intraplaque hemorrhage sensitive (TE 4 ms; TR 670 ms; TI 370 ms, ST 1 mm; matrix size 192 × 256; gap 0 mm; FOV 180 mm; FOV phase 75; Q3 NEX 3; sequence length 5:49 min).
3. T2**-**weighted TSE sequence, axial planes (TE 72 ms; TR 4580 ms; ST 4 mm; matrix size 294 × 384; gap 0.4 mm; FOV 230 mm; FOV phase 100, TF 14; Q3 NEX 2; sequence length 3:18 min).
4. The 3D_TOF (time of flight) sequence, axial planes (TE 7 ms; TR 24 ms; ST 1 mm; matrix size, 198 × 384; gap 0 mm; FOV 200 mm; FOV phase 75%; Q3 NEX 1; sequence length 2:43 min).

### Evaluation of plaque characteristics

In the second phase of the study, the evaluation of carotid plaque characteristics was carried out based on CT, MRI, and DUS examinations. Hounsfield units (HUs) were used to distinguish the character of the plaque as lipid (<60 HU), fibrotic (60–130 HU), mixed (both measured values at <60 HU and 60–130 HU with possible calcification content of no more than 70% of plaque volume), and calcified (>130 HU in more than 70% of the plaque) (17, 18). Additionally, the presence of calcification on a CT examination was evaluated if it appeared to take up 20–30% of the plaque volume. MRI was used to evaluate plaques as lipid (hyposignal on T2-WI), fibrotic (hyper/isosignal on T2-WI), and calcified (marked hyposignal region on all sequences). DUS was utilized to determine plaque characteristics based on echogenicity, including lipid (anechogenic), fibrotic (echogenic), and calcified (hyperechogenic). Additionally, homogeneity (same echogenicity in the entire plaque volume) or heterogeneity (different echogenicity) of the plaque was noted. Finally, the surface of the carotid plaque was evaluated using all of the imaging methods mentioned. Smooth (regular morphology), irregular (irregular morphology and surface), and ulcerated (formed cavities at least 1–2 mm in size) plaque surfaces were also described (17, 19). All plaque features were visually evaluated and manually measured by 2 readers (D.S. and D.P.) in the standard radiological viewer.

Final plaque characteristics were determined by consensus using all three methods (CT, MRI, and DUS). In case of disagreement, the MRI evaluation was used. For the demographical patient data, only the age and sex of the patients and the degree of stenosis together with stenosis location were recorded.

### Statistical analysis

The correlation between carotid stenosis percentage evaluated using CTA and DUS was evaluated using the Spearman’s correlation coefficient (*r*). An estimate for the minimum sample size calculation to determine whether a correlation coefficient differed from zero was based on the expected minimal correlation coefficient *r* = 0.25 with type I error rate α (two-tailed) = 0.05 and type II error rate *β* = 0.20. A prestudy statistical calculation determined that a minimum sample size of 123 patients was required to complete the study. Assuming that 30% of screened patients would not meet all inclusion criteria or complete the study, a minimum of 176 patients were needed to screen for study eligibility.

The normality of the data distribution was checked using the Shapiro–Wilk test. Non-parametric Spearman Rho analysis was used to measure the strength of monotonic association between DUS measurements and CTA evaluation of stenosis severity. To compare statistical differences between particular correlations in different plaque types, Kendall’s rank correlation coefficients with 95% confidence intervals instead of Spearman’s rank correlation were used due to the small-and moderate-sized datasets. Kendall’s rank correlation was a suitable nonparametric technique for such cases. Furthermore, the differences between Tau a and Tau b coefficients showed the variability in associations according to the tied ranks. The nonparametric bootstrap estimation with 1,000 replications was performed while estimating Kendall’s rank correlation coefficients to explore confidence intervals.

Following interpretations of Spearman’s rank correlation and Kendall’s rank correlation were used to provide clear results of their relationship: weak (Spearman’s rho <0.29; Kendall’s tau <0.19), moderate (rho 0.30–0.39; tau 0.20–0.29), strong (rho 0.40–0.69; tau 0.30–0.59), and very strong (rho 0.70–1.00; tau 0.60–1.00). All tests were carried out at a 0.05 alpha level of significance using STATA 17 software (Stata Corp LLC, College Station, TX, USA).

## Results

Of 176 consecutive patients, 143 patients (59.4% males) with ICA stenosis of ≥40% based on DUS B-mode examinations were included. A total of 21 patients were excluded due to contralateral carotid stenosis or occlusion and 12 due to inability to perform CT or MRI examinations (iodine allergy, claustrophobia, patient non-cooperation, and insufficient image quality). All patients underwent CTA, DUS, and MRI examinations. Demographic data are presented in Table 1. We did not notice collapse of ICA lumen in any patient.

**Table 1:** Demographic and imaging characteristics of included patients.

	Screened patients	Enrolled patients
Number; *n*	176	143
Male sex; *n* (%)	110 (62.5)	85 (59.4)
Age (years); mean ± SD	69.6 ± 7.2	69.9 ± 7.3
Degree of stenosis according to CTA (%); mean ± SD	63.5 ± 13.4	74.8 ± 12.5
Side of stenosis – right; *n* (%)	87 (49.4)	69 (48.3)

**Figure 3:**
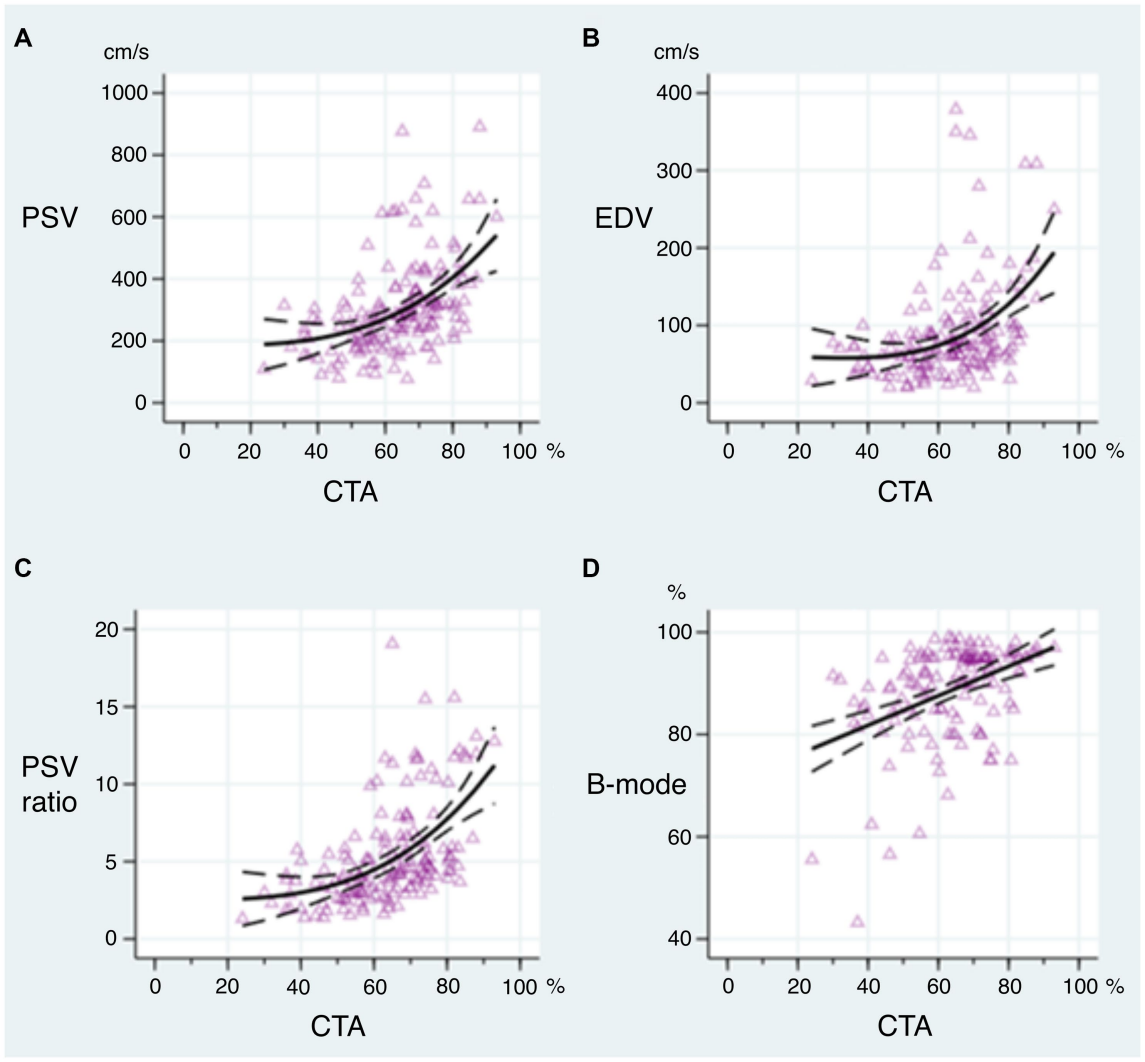
Correlation between peak systolic velocity (PSV) **(A)**, end-diastolic velocity (EDV) **(B)**, PSV_ICA/ICA_ ratio in stenosis and distal to stenosis **(C)**, and B-mode measurement **(D)** with computed tomography angiography (CTA) assessment of internal carotid artery stenosis using exponential **(A–C)** or linear **(D)** generalized linear model with 95% confidential interval.

In the correlation analysis between several DUS techniques and CTA, the PSV_ICA/ICA_ ratio showed the highest correlation with CTA (Spearman’s correlation, rho = 0.576), followed by PSV (rho = 0.526), B-mode (rho = 0.482), and EDV (rho = 0.441; *p* < 0.001 in all cases) – see Table 2 and Figure 3. When comparing the PSV_ICA/ICA_ ratio with other ratios, PSV_ICA_/PSV_CCA_ ratio (rho = 0.482; *p* < 0.001) and PSV_ICA_/EDV_CCA_ ratio (rho = 0.518; *p* < 0.001) showed also weaker correlations with CTA. Tables 2–4 show that all 95% confidential intervals of individual correlations overlap, i.e., correlations between individual DUS methods and CTA are not statistically significantly different at the 5% level of significance.

**Table 2:** Correlation between duplex sonography measurements and computed tomography angiography in evaluation of severity of internal carotid artery stenosis.

	Spearman’s correlation; rho	Kendall’s correlation; tau (95% CI)	*p* value
PSV	0.526	0.360 (0.279–0.450)	<0.001
EDV	0.441	0.300 (0.193–0.408)	<0.001
PSV_ICA/ICA_ ratio	0.576	0.415 (0.316–0.486)	<0.001
B-mode	0.482	0.332 (0.235–0.430)	<0.001

CI, confidence interval; CTA, computed tomography angiography; EDV, end-diastolic velocity; PSV, peak systolic velocity.

Plaque composition analysis characterized 21 plaques as lipid, 50 as fibrous, 66 as mixed, and six as calcified. To increase the statistical power for evaluation of plaque calcifications we added intraplaque calcification to our analysis which was found in 95 cases. The worst correlation was present in the calcified plaque (Kendall’s correlation for PSV, tau = 0.238), where the EDV measurement was less affected by it (tau = 0.523). Measurement of PSV_ICA/ICA_ ratio showed the best correlation with CTA in all plaque types, except plaque with calcifications.

The surface of the plaque was divided into smooth (24 plaques), irregular (84 plaques), and ulcerated (35 plaques) categories. The B-mode measurement correlation was superior on the smooth plaque surface (tau = 0.677 vs. tau = 0.623 for EDV, tau = 0.609 for PSV_ICA/ICA_ ratio, and tau = 0.584 for PSV), and the PSV_ICA/ICA_ ratio was best correlated for irregular (tau = 0.373 vs. tau = 0.330 for PSV, tau = 0.282 for B-mode, and tau = 0.229 for EDV) and ulcerated (tau = 0.382 vs. tau = 0.309 for EDV, tau = 0.306 for PSV, and tau = 0.279 for a B-mode) plaque surfaces (Table 3).

**Table 3:** Correlation in evaluation of severity of internal carotid artery stenosis measured using Kendall’s rank correlation coefficient between duplex sonography measurements and computed tomography angiography based on arterial diameter reduction in different plaque types.

	PSV vs. CTA tau (95% CI); *p* value	PSV_ICA/ICA_ ratio vs. CTA tau (95% CI); *p* value	EDV vs. CTA tau (95% CI); *p* value	B-mode vs. CTA tau (95% CI); *p* value
*Plaque type*				
Lipid plaque; *n* = 21	0.398 (0.284–0.516) <0.001	0.406 (0.280–0.499) <0.001	0.329 (0.170–0.420) <0.001	0.351 (0.222–0.480) <0.001
Fibrous plaque; *n* = 50	0.318 (0.136–0,476) <0.001	0.461 (0.274–0.600) <0.001	0.304 (0.137–0.491) <0.001	0.350 (0.172–0.527) <0.001
Mixed plaque; *n* = 66	0.396 (0.260–0.537) <0.001	0.403 (0.251–0.513) <0.001	0.308 (0.149–0.448) <0.001	0.300 (0.136–0.463) <0.001
Calcified plaque; *n* = 6	0.238 (−0.724–0.946) 0.794	0.333 (−0.520–0.964) 0.557	0.523 (−0.246–1.000) 0.230	0.390 (−0.379–1.000) 0.320
*Intraplaque calcifications*				
Intraplaque calcification; *n* = 95	0.342 (0.231-0.482) <0.001	0.396 (0.283-0.502) <0.001	0.280 (0.142-0.391) <0.001	0.287 (0.146-0.451) <0.001
*Plaque surface*				
Smooth surface; *n* = 24	0.584 (0.317–0.792) <0.001	0.609 (0.361–0.827) <0.001	0.623 (0.390–0.813) <0.001	0.677 (0.514–0.839) <0.001
Irregular surface; *n* = 84	0.330 (0.200–0.440) <0.001	0.373 (0.243–0.454) <0.001	0.229 (0.101–0.344) <0.001	0.282 (0.152–0.413) <0.001
Ulcerated surface; *n* = 35	0.306 (0.167–0.554) <0.001	0.382 (0.191–0.585) <0.001	0.309 (0.061–0.510) 0.013	0.279 (0.042–0.515) 0.021

For carotid stenosis measurement using area reduction, the PSV_ICA/ICA_ ratio was best correlated regardless of plaque type or surface, except for calcified plaque. Detailed data are shown in Table 4.

**Table 4:** Correlation in evaluation of severity of internal carotid artery stenosis measured using Kendall’s rank correlation coefficient between duplex sonography measurements and computed tomography angiography based on area reduction in different plaque types.

	PSV vs. CTA tau (95% CI); *p* value	PSV_ICA/ICA_ ratio vs. CTA tau (95% CI); *p* value	EDV vs. CTA tau (95% CI); *p* value	B-mode vs. CTA tau (95% CI); *p* value
*Plaque type*				
Lipid plaque; *n* = 21	0.416 (0.292–0.538) <0.001	0.456 (0.314–0.549) <0.001	0.384 (0.217–0.446) <0.001	0.284 (0.137–0.432) <0.001
Fibrous plaque; *n* = 50	0.319 (0.120–0.486) 0.001	0.464 (0.267–0.304) <0.001	0.288 (0.125–0.463) 0.001	0.286 (0.108–0.464) 0.002
Mixed plaque; *n* = 66	0.456 (0.325–0.599) <0.001	0.498 (0.331–0.616) <0.001	0.391 (0.219–0.519) <0.001	0.259 (0.065–0.452) 0.009
Calcified plaque; *n* = 6	0.048 (−0.746–0.689) 0.939	0.142 (−0.599–0.768) 0.809	0.333 (−0.306–0.813) 0.375	0.195 (−0.656–1.000) 0.653
*Intraplaque calcifications*				
Intraplaque calcification; *n* = 95	0.344 (0.234–0.481) <0.001	0.399 (0.287–0.504) <0.001	0.285 (0.151–0.388) <0.001	0.294 (0.170–0.417) <0.001
*Plaque surface*				
Smooth surface; *n* = 24	0.587 (0.316–0.773) <0.001	0.655 (0.367–0.850) <0.001	0.650 (0.440–0.804) <0.001	0.618 (0.419–0.817) <0.001
Irregular surface; *n* = 84	0.378 (0.254–0.500) <0.001	0.431 (0.296–0.524) <0.001	0.264 (0.141–0.377) <0.001	0.261 (0.125–0.396) <0.001
Ulcerated surface; *n* = 35	0.234 (0.053–0.551) 0.018	0.301 (0.116–0.566) 0.003	0.271 (0.021–0.508) 0.034	0.055 (−0.211–0.322) 0.684

## Discussion

The results of the present study showed that all of the US methods we used for the assessment of carotid artery stenosis strongly correlated with CTA and might be used in clinical practice. The PSV_ICA/ICA_ ratio showed the highest correlation, followed by PSV, B-mode, and EDV. All correlations were affected by plaque composition and plaque surface, whereas calcified plaques made an accurate measurement of stenosis very difficult. When the plaques were soft (lipid or fibrous) and/or had a smooth surface, the correlation between methods was the highest. A very strong correlation was found between all DUS measurements when correlated with CTA, except PSV, when the plaques had a smooth surface. A strong correlation occurred for all other plaque characteristics, except calcified plaques where weak correlations were detected, and plaques with intraplaque calcifications, irregular or ulcerate surface where predominantly moderate correlations were detected.

DSA may still be considered the gold standard for measuring carotid artery stenosis, but it has been gradually replaced by available noninvasive and semi-invasive imaging methods, such as CT, DUS, and MRI over the last few decades (11). CTA was selected as the gold standard for comparison in the present study because it is a widely available and noninvasive imaging method when compared to DSA. Dedicated software was used to accurately measure stenosis to avoid the effect of operator bias on the accuracy of manually measured degree of stenosis.

Among the mentioned diagnostic methods, DUS remains the primary noninvasive imaging method for detecting, classifying, and monitoring carotid stenosis due to its widespread availability, low cost, and high resolution (20). US techniques have demonstrated high accuracy before becoming acceptable and precise imaging methods in order to avoid DSA, which is an invasive and potentially harmful procedure (21). However, the current guidelines only mentioned PSV or EDV as the initial diagnostic imaging method of choice to evaluate the severity of stenosis in symptomatic and asymptomatic patients (22), while the B-mode was recommended only as an adjunctive method (23). The present study results confirmed that PSV correlated better with the gold standard CTA compared to the B-mode. Nevertheless, PSV may still be compromised in many clinical situations (e.g., atrial fibrillation, low cardiac ejection fraction, severe heart valve disease, and contralateral ICA occlusion). To avoid measurement bias or inaccuracy, the PSV_ICA/ICA_ ratio might be used as an additional measurement of carotid stenosis severity. However, for the PSV ratio, it is necessary to measure the PSV proximal to the stenosis or sufficiently distal to the stenosis where flow normalization already occurs. As the ICA stenosis is in most cases in the origin of ICA, it is not possible to measure PSV proximal to the stenosis in the ICA. And, flow in the mid-section of the ICA several centimeters distal to the stenosis often cannot be visualized with a linear probe, as the artery is located at a greater depth and visualization is hindered by the mandible.

The PSV ratio is commonly used to evaluate arterial stenosis in various arteries in the human body, including vertebral (24, 25), peripheral (26), or renal (27) arteries, where it is superior to other Doppler parameters. In the present study, the PSV_ICA/ICA_ ratio showed the highest correlation with CTA in evaluation of carotid stenosis severity. Nevertheless, the differences between correlations using different sonographic parameters were not statistically significant. Based on these results, it can be concluded that the PSV_ICA/ICA_ ratio should be included among the commonly used parameters for the evaluation of carotid stenosis. These results are in concordance with a recently published study (28).

The study results also showed that US techniques overestimated the degree of stenosis compared to CTA. The results of other studies are heterogeneous, as some of them confirm the present study results with respect to DUS (29, 30), while others show that DUS underestimates carotid stenosis compared to CTA (31). Correlation analysis of US techniques and CTA demonstrated results (moderate correlation) that were similar to those from a prior study (32). Another study has found that CT underestimated and DUS overestimated the degree of stenosis when compared to measurements obtained using DSA (33). Overestimation using DUS might be due to hemodynamically significant contralateral carotid stenosis or occlusion (34, 35). However, blood flow velocities are also affected by residual lumen shape, length of stenosis or other hemodynamic factors (36, 37). On the other hand, misrepresentation of calcification (38) or diameter-based measurements (30, 39), particularly in an irregularly shaped lumen (40), are associated with significant underestimation of stenosis by CT. Therefore, together with diameter-based measurement, stenosis evaluation using area reduction was also used in the present study and these correlations showed higher values (presented in Tables 3, 4).

Carotid atherosclerotic plaque characteristics belong to the group of other factors that potentially affect the precision of carotid stenosis measurement. To the best of our knowledge, this is the first study to investigate the relationship between plaque features and carotid stenosis measurement accuracy. Calcified plaques had a negative impact on the accuracy of carotid stenosis evaluation by all DUS techniques. Study results also showed that evaluation using PSV was even more affected by calcifications compared to B-mode measurement. However, the results were based only on limited numbers of calcified plaques found in patients in our cohort. Nevertheless, subanalysis results showed that only a large volume of plaque calcifications negatively affected the accuracy of stenosis measurement. This could be explained by acoustic shadowing on DUS examinations caused by large calcifications, leading to inaccurate determination of the degree of carotid stenosis far below the level needed for clinical decision-making (41).

Lipid, fibrotic, or mixed plaques showed a good correlation between CTA and all used DUS techniques. However, the PSV_ICA/ICA_ ratio provided the highest correlations. In terms of plaque surface type, the best correlation was present between DUS and CTA in plaques with a smooth surface. The level of correlation became moderate with an irregular and ulcerated surface. For stenosis evaluation in B-mode, the correlations were significantly worse in plaques with irregular and ulcerated surfaces compared to smooth plaques.

Several study limitations should be mentioned. First, CTA was used as the gold standard for carotid stenosis evaluation. However, studies comparing carotid stenosis evaluation using CTA and DSA or CTA and histological specimens showed that CTA and even DSA underestimated the percentage of stenosis by approximately 12% (30). Thus, to identify the most accurate protocol for evaluation of carotid artery stenosis severity, all available imaging methods and all suitable sonographic parameters with additional histopathological plaque evaluation needed to be included and compared to determine the influence of plaque characteristics on the accuracy of stenosis measurement. Second, MRI, CTA, and DUS were used for evaluation of plaque composition and plaque surface instead of the histopathological evaluation of the plaque specimens. Moreover, only manual evaluations and visual measurements were made without using any digital image analysis. Third, imaging of distal extracranial ICA and measurement of the velocities through the submandibular window could be more difficult to perform in high-grade stenosis and near occlusion. Nevertheless, we have not experienced any problem in any patient with these measurements. Fourth, the measurement of carotid stenosis with calcified plaques in the B-mode could be influenced by artifacts from calcified plaques. However, we were able to measure residual lumen in all study patients with calcified plaques when using different approaches on the neck. Fifth, the sample size was calculated for the expected minimal correlation coefficient *r* = 0.25 and did not allow for validation of the PSV, EDV, and PSV_ICA/ICA_ ratio for individual ranges of carotid stenosis. And, it was performed only for the overall correlation of individual methods, not for individual subtypes of atherosclerotic plaques. Thus, a follow-up study including patients indicated for carotid endarterectomy that would allow for histopathological evaluation of plaque specimens with DUS, CTA, and MRI examinations prior to intervention is planned. We plan also to establish cut-off values for different severity of carotid stenosis, for individual age ranges and genders.

### Clinical implications

In patients with ICA stenosis consisting of a non-calcified plaque with a smooth surface, B-mode measurements, EDV, local PSV measurements and/or the PSV_ICA/ICA_ ratio can be used potentially to evaluate the stenosis with DUS as the correlations with CTA were strong to very strong in all cases without statistically significant differences. In patients with calcified plaques or plaques with an irregular or ulcerated surface, it is appropriate to use the PSV_ICA/ICA_ ratio, because the correlations with CTA were strong in all these cases, while the local measurement of PSV, EDV, or measurements in the B-mode achieved only a moderate to strong correlation.

## Conclusion

The PSV, PSV_ICA/ICA_ ratio, EDV, and B-mode measurements showed comparable correlation in evaluation of carotid artery stenosis as measured by their correlation with CTA. US measurement method comparison revealed that the PSV_ICA/ICA_ ratio had a better correlation compared to PSV or B-mode measurement, although this result was not statistically significant. Carotid artery stenosis evaluation using DUS and CTA examinations was significantly affected by plaque composition and plaque surface. Irregularity or ulceration negatively influenced the measurement, whereas no correlation was found for calcified plaque.

## Data availability statement

The raw data supporting the conclusions of this article will be made available by the authors, without undue reservation.

## Ethics statement

The studies involving humans were approved by Ethics Committee of University Hospital Ostrava. The studies were conducted in accordance with the local legislation and institutional requirements. The participants provided their written informed consent to participate in this study.

## Author contributions

DP: evaluation of plaque characteristics, article writing, funding, and project coordination. AV: evaluation of plaque characteristics and article writing. TJ: CTA examination and images evaluation. MR and MK: US examination. VC: CTA examination and images evaluation. RH: article review and US examination. TH: statistics. DŠ: supervision, US examination, article review, and project coordination. All authors contributed to the article and approved the submitted version.
